# Effect of Different Reduced Training Frequencies After 12 Weeks of Concurrent Ballistic and Aerobic Training on Muscle Power and Triceps Brachii Muscle Architecture

**DOI:** 10.3390/jfmk10010037

**Published:** 2025-01-19

**Authors:** Thomas Mpampoulis, Spyridon Methenitis, Angeliki N. Stasinaki, Nikolaos Zaras, Gregory C. Bogdanis, Gerasimos Terzis

**Affiliations:** 1 Sports Performance Laboratory, School of Physical Education & Sports Science, National and Kapodistrian University of Athens, Daphne, 17237 Athens, Greece; smetheni@phed.uoa.gr (S.M.); agstasin@phed.uoa.gr (A.N.S.); gbogdanis@phed.uoa.gr (G.C.B.); 2 Department of Physical Education and Sport Science, Democritus University of Thrace, 69100 Komotini, Greece; nzaras@phyed.duth.gr; 3 Human Performance Laboratory, Department of Life Sciences, School of Life and Health Sciences, University of Nicosia, Nicosia 2417, Cyprus

**Keywords:** detraining, muscle hypertrophy, cardiovascular endurance, high-intensity training, interference effect, concurrent training effect

## Abstract

**Background/Objectives:** The aim of the present study was to investigate the effect of two long-term reduced concurrent training frequencies (incorporating power training for the upper and high-intensity interval aerobic training for the lower extremities), in which participants performed one training session every either 7 or 14 days, after 12 weeks of systematic concurrent training on upper extremities’ muscle strength, power, and morphology in young females. **Methods:** After a 12-week concurrent resistance and aerobic training period, participants were assigned into three groups and performed either one training session every 7 days (G7), or once every 14 days (G14), or detraining (GD) for 12 weeks, followed by 12 additional weeks of detraining. **Results:** Performance and muscle mass increased after the initial 12-week training period. After the reduced training frequency period, bench press 1-RM and aerobic power remained unchanged in G7 and decreased significantly in G14 (−5.9 ± 4.9%; −1.4 ± 4.5%). Muscle power and muscle thickness of the triceps branchii long head decreased significantly in G7 (−9.8 ± 7.7%; −0.9 ± 0.6%; respectively, *p* < 0.05) and G14 (−10.9 ± 7.6%; −2.8 ± 2.7%, respectively, *p* < 0.05), without significant differences between groups (*p* > 0.05). **Conclusions:** In conclusion, 12 weeks of systematic concurrent resistance (upper extremities) and aerobic training (lower extremities) induced significant improvements in upper extremities muscle power/strength and muscle architecture characteristics. Both reduced training frequencies led to significant reductions in power performance. Thus, performing one training session every 2 weeks for 3 months may preserve 90 to 95% of the muscle power/strength, aerobic power and 72% of muscle mass adaptations achieved with systematic concurrent training. However, greater preservations in the above parameters could be observed if the training frequency is one training session per week.

## 1. Introduction

Various organizations such as The American College of Sports Medicine recommend a training regimen that incorporates both resistance and aerobic exercises. The latest exercise recommendations indicate that adults should engage in a minimum of 150 min of moderate-intensity cardiorespiratory exercise each week, along with resistance training targeting all major muscle groups at least twice weekly, to improve and sustain physical fitness and overall health [1]. A combination of resistance and aerobic training is widely adopted by numerous fitness enthusiasts. Prolonged engagement in an exercise that incorporates both resistance and aerobic training leads to enhancements in muscle strength, power and mass, and cardiovascular endurance, alongside advancements in metabolic function and mental well-being [2,3,4]. These adaptations are crucial for improving/maintaining health and improving overall performance in individuals, regardless of their age, gender, or health condition [3,5], based on the aforementioned research results. Performing concurrent resistance training (strength or power training) and aerobic training on the same training day, for many training enthusiasts, is an integral part of their training routine to improve both their body performance and health [6]. Indeed, it is well established that engaging, especially, in high-intensity concurrent resistance and cycling endurance training twice per week over several weeks can lead to substantial improvements in body composition, muscle strength, mass, and aerobic capacity in young females [7,8].

While the advantages of consistent, long-term physical activity for health and fitness are broadly recognized, a notable number of individuals frequently decrease or abandon their training efforts only a few months after commencing a structured exercise plan [9,10]. A decrease in training frequency or the total cessation of exercise, known as detraining, leads to a progressive decline in some or all adaptations that gained through the systematic training period [11]. Indeed, just after 4 to 6 weeks, notable decreases occur in muscle power/strength [12,13,14], muscle mass [15,16], and cardiovascular performance [17,18]. Given the considerations outlined above, it is crucial to acknowledge that a significant challenge in modern times lies not only in achieving notable improvements in physical fitness and health through short-term systematic training programs but also in preventing the loss of these improvements after a brief period of detraining, including those related to body composition, muscle strength/power, cardiorespiratory fitness, and lipidic/glycemic blood profiles. Therefore, it is of significant clinical and practical importance to investigate the minimum training frequency that it is necessary to sustain much of these adaptations during periods of forced reduced training frequency. It seems that the training-induced adaptations were observed after systematic training, including muscular strength [19,20,21,22,23], lower extremities hypertrophy [24], and aerobic performance [25,26], could at least partly be preserved if individuals perform two or even one training session/s per week over a short-termed period [20]. Furthermore, it has been recently reported that training once per week, with concurrent training including strength and aerobic training for the same muscle groups, leads to a maintenance of training-induced adaptations after a period of 12 weeks of reduced training frequency [7]. Furthermore, even a training frequency of one training session every 2 weeks, could lead to preservation of 90 to 95% of the muscle mass/strength and aerobic power adaptations achieved during the systematic training period [7]. It seems that the key parameters for these observations are the maintenance of the training intensity and/or volume during the period of forced reduced training frequency, to comparable levels like those applied at the end of the regular training phase [7,23,25]. However, these conclusions are based on studies investigating the effect of the reduced training frequency after either resistance (strength/hypertrophy training) or aerobic training alone, or concurrent resistance (strength/hypertrophy training) and aerobic training performed in the same muscle groups, and over a short period of detraining or reduced training frequency. Thus, until now, it is unknown whether long-term reduced training frequency of concurrent resistance and aerobic training on different muscle groups may preserve the concurrent training-induced adaptations in both neuromuscular and aerobic performances. Furthermore, until now it remains unclear whether infrequent power training, whether performed alone or in combination with endurance training targeting the same or different muscle groups, can effectively preserve the adaptations induced by regular power training. The question revolves around whether such an infrequent training approach can serve as a sufficient stimulus to maintain the benefits of power training during times of reduced training volume or frequency. Thus, the main aim of the present study was to investigate the effect of two long-term reduced concurrent training frequencies (incorporating power training for the upper and HIIT aerobic training for the lower extremities) in which participants performed one training session every either 7 or 14 days, after 12 weeks of systematic concurrent training on upper extremities’ muscle strength, power, and morphology in young females. Secondly, to examine how the above training frequencies could affect the magnitude of training-induced adaptation losses during a subsequent period of detraining. It was hypothesized that training every 7 days during the reduced training period would preserve most of the muscle mass and strength/power adaptations while training every 14 days would result in a greater loss of muscle strength/power and mass adaptations. Moreover, the detraining period would have the same impact on the losses in any adaptations that were preserved with both reduced training modalities.

## 2. Materials and Methods

### 2.1. Experimental Approach

**Figure 1:**
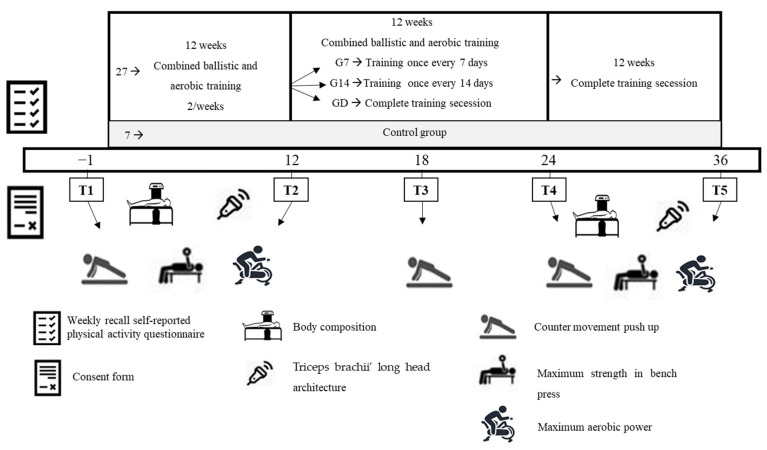
The experimental design of the present study.

Participants were recruited via advertisements in university student societies. Responders visited the laboratory, where they completed a weekly recall self-reported physical activity questionnaire to verify if the inclusion criteria were met. Female students who met the inclusion criteria (only healthy females, aged between 18 and 26, with no systematic experience in resistance/ballistic or aerobic training for at least 2 years) visited the laboratory for a second time three days later for a body height, body composition, and the muscle architecture of triceps brachii long head evaluation. After the above evaluations, they performed a familiarization session for all the performance evaluations. Then, during their third and fourth visits (two days after), all the evaluations were performed. Thereafter, participants were randomly allocated into two groups [based on their upper extremities muscle strength, (no differences between groups; *p* > 0.05)]: (A) training group: participants performed a 12-week combined ballistic (power) and aerobic training (two training sessions per week) and (B) control group (GC) who did not perform any kind of training. After this period, participants of the training group were divided into three sub-groups and for the next 12 weeks performed either one training session per week (G7), or one training session per 14 days (G14) or full detraining (GD; Figure 1). The allocation of the participants into the three sub-groups was performed based on the post-training values of their upper extremities’ muscle strength (no difference between groups; *p* > 0.05). After the end of the second 12-week period, participants followed a 12-week period of complete training cessation. The evaluations of body composition, muscle architecture of triceps brachii long head, bench press maximum strength (1-RM), countermovement push up (CMPU), and cycling maximal aerobic power were performed, with the same order and at the same conditions, one week before (T1) and after (T2) the systematic training period, as well as one week after the end of the reduced training (T4) and detraining (T5) periods. An additional evaluation of CMPU was performed 6 weeks after the initiation of the reduced training period (T3) to identify a potential difference in the rate of possible power performance changes between the groups (Figure 1).

### 2.2. Participants

Based on the design of the present study and data from the pilot study, power analysis revealed that a total of 30 individuals had to participate and complete the study for an actual power of 0.900 for the results of the present study (G*Power, Version 3.1.9.4). Thirty-four healthy, female physical education students (age: 20.03 ± 2.1 years, body mass: 60.9 ± 5.6 kg, body height: 164.1 ± 4.5 cm) participated and completed the study. The participants were in good health with no musculoskeletal injuries, did not receive nutritional supplements, and did not have any experience with systematic ballistic or aerobic training for at least 2 years prior to the initiation of the present study. All the participants signed an informed consent clearly stating that they were free to withdraw from the study at any time point without stating the reason. All the procedures were according to the Declaration of Helsinki as revised in 2000 and were approved by the approved by the Ethics Committee of the School of Physical Education & Sports Science, the National and Kapodistrian University of Athens (number1372/20-04-2022).

### 2.3. Procedures

#### 2.3.1. Concurrent Resistance and Aerobic Training

During the period of systematic training (T1 to T2), all participants in the training group (N = 27) performed a concurrent ballistic (in upper extremities) and aerobic (in lower extremities) training session (in exactly that order), two times per week, with at least 72 h between sessions. The specific order of the two training stimuli was adapted, based on previous studies reporting that when resistance training is performed before aerobic training (HIIT), the adaptations in muscle strength and mass are not attenuated, while the opposite order seems to favor concurrent training effect [27,28]. Each training session lasted ~1 h and started with a 5 min warm-up on a bicycle at 50 Watts followed by 5 min of stretching for the upper and lower extremities. Then, they performed, for warm-up, one set of 12 repetitions at 30% of 1-RM and one set of 6 repetitions at 40% of 1-RM bench presses on a smith machine. During the first week of training, the main training part was composed of three sets of six repetitions at 40–50% 1-RM of ballistic bench presses on the smith machine, to avoid any extensive muscle damage due to the unaccustomed activity. Then, during the second week, the external load increased up to 60% of 1-RM. After the second week, the external load was increased by 2.5–5% each week. Ten minutes after the end of the above training module, participants performed their endurance training on a stationary bicycle (50 revolutions per minute dictated by a metronome) [8,29]. During the first training week, participants performed five sets of 60 s at 100% of their maximal aerobic power. During the first training session of the second week, they performed seven sets of 60 s at 100% of their maximal aerobic power, which increased to eight sets of 60 s in the second training session. Thereafter, all the participants performed 10 sets of 60 s at 100% of their maximal aerobic power. The passive recovery between the sets was 60 s. The cycling workload (Watts) was increased by 2.5–5% in every training session [8,29]. The participants of the GC did not follow any type of systematic training throughout the study. The rate of perceived exertion [30] was evaluated after the end of the second training session of each week (RPE, CR10 scale: 0: very very light to 10: very very heavy).

#### 2.3.2. Reduced Training and Complete Cessation of Exercise

After the end of the initial 12-week training period (T1 to T2), participants of the training group were assigned into three sub-groups and continued for another 12 weeks (T2 to T4) as follows: (A) 10 participants performed the same concurrent training once per week (G7; n = 10), (B) 10 participants performed the same concurrent training every 14 days (G14; n = 10), while the remaining seven participants followed a complete training secession (GD; n = 7). The G7 and G14 groups continued their training with the training intensities and volumes that were used at the last training session of the regular training period, which was maintained in both groups until the end of the reduced training frequency period (T4). After the end of the reduced training frequency period, all participants followed a 12-week period of complete training cessation (T4 to T5)

#### 2.3.3. Evaluation of Body Composition

Participants reported at the laboratory during morning hours at a fasted state for the evaluation of their body composition through dual-energy x-ray absorptiometry (Prodigy Pro, General Electric, Madison, WI, USA). They were asked to abstain from strenuous physical activities for at least 24 h before the evaluation. All the measurements were analyzed using the Lunar encore v.18 software for the determination of total and upper extremities’ lean body mass (LBM) and fat mass. The intraclass correlation coefficients (ICCs) for body mass, fat mass, and LBM were 0.99 for all the variables.

#### 2.3.4. Performance Evaluations

*Countermovement push-up test.* The muscle power of the upper extremities was evaluated through a countermovement push-up test (CMPU). The test was performed on a force platform (Applied Measurements Ltd., Co., Aldermaston, UK, WP800 80 × 80 cm, sampling frequency 1 kHz). After a 5 min warm-up on a stationary bicycle at 50 Watts and one set of five push-ups, participants performed three CMPUs with submaximal intensity and then three maximal CMPUs with 2 min rest between each repetition. Marks were placed on the force-platform on which participants were required to position their hands. The distance between the marks was equivalent to the participant’s shoulder-width. The distance between the marks was recorded and used during the re-evaluations. Participants always carried out the tasks by resting their hands on the force-plate, while the remaining supports (feet/knees) were positioned on another rigid surface placed at the same level as the force-plate. During the test, participant’s weight was evenly distributed to the force plate beneath each hand. The shoulders were placed at 90 degrees of flexion, with the torso, legs, and elbows fully extended. Following a 3 s countdown, the participant immediately lowered their torso rapidly toward the force plate, then they immediately performed a CMPU. The whole countermovement phase was performed as fast as it could be. Prior to each attempt, they were instructed to perform their maximum, and to achieve the highest height that was possible. During the landing, they had been instructed to try to “soften the hands” upon impact. Each effort was recorded and analyzed (Kyowa sensor interface PCD-320A) in order to calculate push up height [((0.5 × flight time) 2 × 2-1) × 9.81] and maximum power [(body weight + Fmax) × 9.81 × flight time] [31,32]. The signal was filtered using a secondary low-pass Butterworth filter with a cutoff frequency of 10 Hz. The best performance according to the push power was used for further analysis. Intersession reliability was determined by comparing the analysis of the data obtained by 10 participants on two separate days, 7 days apart. Participants did not perform any strength training one week prior to first data-collection and through the whole evaluation. Participants performed the procedure in the same hours and with the same sequence on both occasions, following exactly the same instructions as discussed above. The ICCs for push-up height and power were 0.88 and 0.94, respectively.

*Bench press 1-RM.* The evaluations of upper extremities’ maximum strength were performed through a bench press exercise in a Smith machine as previously described [33,34]. Thirty minutes after the evaluation of the CMPU performance, participants performed one set of 10 repetitions with approximately 40% of the predicted 1-RM for warm up. Then, they performed three sets of eight, four, and two repetitions with approximately 50–60%, 70–75%, and 80–85%, respectively, of their predicted 1-RM. Thereafter, 3–6 sets of single repetitions were performed for the determination of their maximum strength. Three minutes of rest was allowed between the sets while two of the researchers were present to supervise the technique of the exercise and to encourage the participants to perform their best. The rate of perceived exertion (CR10 scale: 0: very very light to 10: very very heavy) [30] was recorded after each set. The ICC of this evaluation is 0.96 in our laboratory [33,34].

*Maximum aerobic power.* Maximum aerobic power was evaluated on a bicycle leg ergometer (Monark 834E; Monark Exercise AB, Vansbro, Sweden) 2 days after the evaluations of CMPU and 1-RM performances. The test was based on the adjusted YMCA Cycle Ergometer Protocol [35] as it has been previously used in our laboratory [8,29]. Specifically, after 5 min of static and dynamic stretching for the lower extremities, the participants started to cycle at 25 Watts with 50 revolutions per minute. The increase in resistance in the second 3 min stage depended on the first-stage heart rate (HR): if HR was under 80 beats per minute (b/min) at the end of the first stage, the resistance in the second stage was increased 100 Watts; if HR fluctuated between 80 and 90 b/min, the resistance was increased 75 Watts; if HR fluctuated between 90 and 100 b/min, the resistance was increased 50 Watts; and if HR was over 100 b/min, the resistance was increased 25 Watts. From the third 3 min stage and on, the resistance was increased by 25 Watts until exhaustion (when each participant could no longer maintain the 50 revolutions per minute). Heart rate was monitored and recorded throughout the test (Polar Α300, Polar Electro, Kampele, Finland). The rate of perceived respiratory and leg exertion was evaluated at the end of each 3 min stage through a CR10 scale (0: very very light to 10: very very heavy) [30]. After the end of the test, the participants continued pedaling for 3–5 min at 25 Watts. The maximum aerobic power and maximum heart rate were defined as those achieved during the last completed stage [8,29]. The ICC for this test is 0.91 in our laboratory [8,29].

#### 2.3.5. Ultrasonography

**Figure 2:**
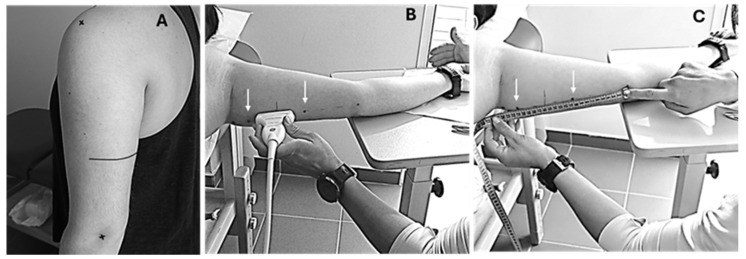
Representative images of the methodology that was followed for the evaluation of triceps brachii long head muscle architecture: (**A**) Marked points representing 60% of the total distance between the acromion and the lateral epicondyle of the humerus while participants were in standing position, (**Β**) transducer alignment parallel to the triceps long head’s fascicles for the evaluation of its architecture properties, using the extended field of view mode (with white arrows representing the area around the 60% of the total distance between the acromion and the lateral epicondyle of the humerus that the largest continuous fascicle was located), while participants were in supine position, and (**C**) measuring and documenting the distance of the marks for reference in future measurements.

**Figure 3:**
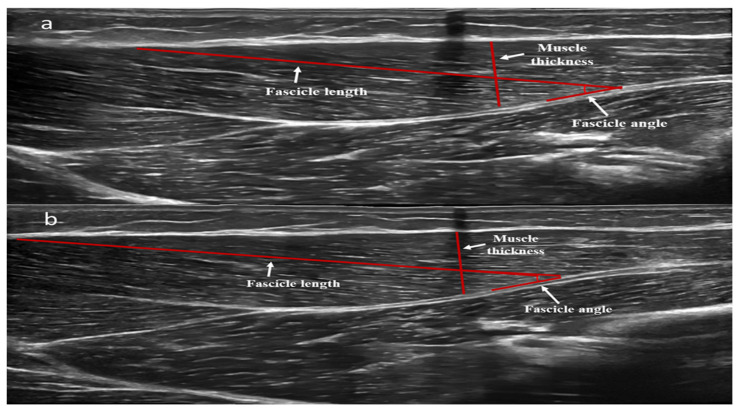
Representative image of the triceps brachii long head muscle architecture taken with an ML6-15 MHz linear-array probe with the extended field of view mode and its analysis to define muscle thickness, fascicle angle, and fascicle length before (**a**) and after (**b**) 12 weeks of systematic concurrent training.

For the measurements of the triceps brachii long head, B-mode axial-plane ultrasound images (LOGIQ S9, General Electric, Boston, MA, USA) were taken with an ML6-15 MHz linear-array probe with the extended field of view mode. Participants were placed at a standing position with their arms fully extended on the side of their body. The posterior surface of the acromion and the lateral epicondyle of the humerus were marked and the distance between them used as the total length of the upper arm (Figure 2) [36]. A marking point was placed at 60% of the distance, starting from acromion. Then, the subjects lay supine with their measuring arm extended on a laboratory bed at a position of 90° to their torso. A dashed line was drawn from the insertion of the triceps brachii long head up to the medial epicondyle of the humerus. The transducer was placed along the dashed line and oriented in parallel to the muscle fascicles. The transducer’s alignment was considered appropriate when several fascicles could be easily outlined without interruption across the image. Based on this orientation, a line (~10 cm) was drawn on the left and the right of the point of 60% to identify and capture the largest continuous fascicle visualization [33,37]. To obtain the muscle image, a continuous single view was taken by moving the transducer along the marked line. Thus, after obtaining the images, two dots were marked on the skin, one on the left edge of the marked line and one on the right edge. Coordinates of each edge of this dashed line were used to warrant the same measurement regions after every time period. Images were analyzed by image analysis software (ImageJ Version 1.54d, National Institutes of Health) for muscle thickness, the fascicle angle, and fascicle length. Muscle thickness was defined as the distance between the superficial and deep aponeurosis, fascicle angle as the angle of insertion of muscle fascicles onto the deep aponeurosis, and fascicle length as the fascicular path between the insertions of the fascicle onto the upper and deeper aponeurosis (Figure 3). The ICC for the measurement of muscle thickness was 0.984, for the fascicle angle was 0.858, and for fascicle length was 0.794.

### 2.4. Statistical Analysis

All data are presented as means and standard deviation (±SD). The Shapiro–Wilks test was used to check the normality of the data. Νo violation was found. Student’s T-tests were used to detect the differences from pre- to post-systematic training values for the initial training period (T1 to T2). A two-way repeated analysis of variance (ANOVA) was used to test the interaction between groups and time (T1 to T5). Bonferroni post hoc analysis was used when it was necessary. Effect sizes were calculated (Hedges’ g). The level of statistical significance was set at *p* ≤ 0.05. The statistical analyses were performed with SPSS version SPSS 25.0 (SPSS Inc., Chicago, IL, USA).

## 3. Results

### 3.1. Systematic Concurrent Training (T1 to T2)

**Figure 4:**
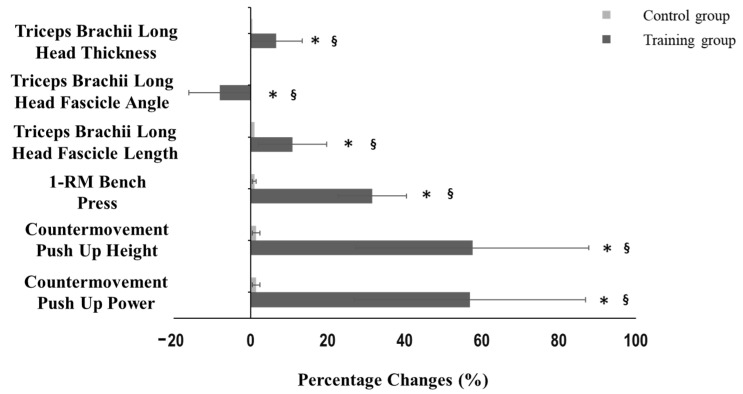
Percentage changes in muscle power in countermovement push-up, height in countermovement push-up, bench press 1-RM, triceps brachii long head fascicle length, fascicle angle, and thickness after 12 weeks of systematic training (T2) in the training and the control group. * *p* < 0.05 significant difference after training, § differences between groups after 12 weeks of systematic training.

Significant increases in the lean body mass of upper extremities (7.3 ± 5.6%, *p* < 0.001, Hedges’ g = 0.600; Table 1), CMPU power and height (57 ± 30.8%, *p* < 0.001, Hedges’ g = 0.688; 57.6 ± 30.2% *p* < 0.001, Hedges’ g = 1.072; respectively, Table 1; Figure 4), bench press 1-RM (31.6 ± 8.9%, *p* < 0.001, Hedges’ g = 2.833; Table 1; Figure 4), maximal aerobic power (20.4 ± 10.3%, *p* < 0.001, Hedges’ g = 1.077; Table 1), HR at 100 and 125 Watts (10.1 ± 6.9%, *p* < 0.001, Hedges’ g = 1.084 and 10 ± 4.1%, *p* < 0.001, Hedges’ g = 1.685; respectively, Table 1) were found after the T1-T2 training period in the training group, with significant difference compared to the control group. Total body fat and total lean body mass remained unchanged for both groups between T1-T2 (*p* > 0.05; Table 1).

Fascicle length and muscle thickness were significantly increased from T1 to T2 in the training group (10.1 ± 8.9%, *p* < 0.001, Hedges’ g = 0.704; 10.7 ± 3.3%, *p* < 0.001, Hedges’ g = 1.000; respectively, Table 1; Figure 4), while fascicle angle was significantly decreased (7.9 ± 4.1%, *p* < 0.001, Hedges’ g = 0.704; Table 1; Figure 4). The above parameters remained unchanged in the control group.

### 3.2. Reduced Training Frequency and Detraining (T2 to T5)

**Figure 5:**
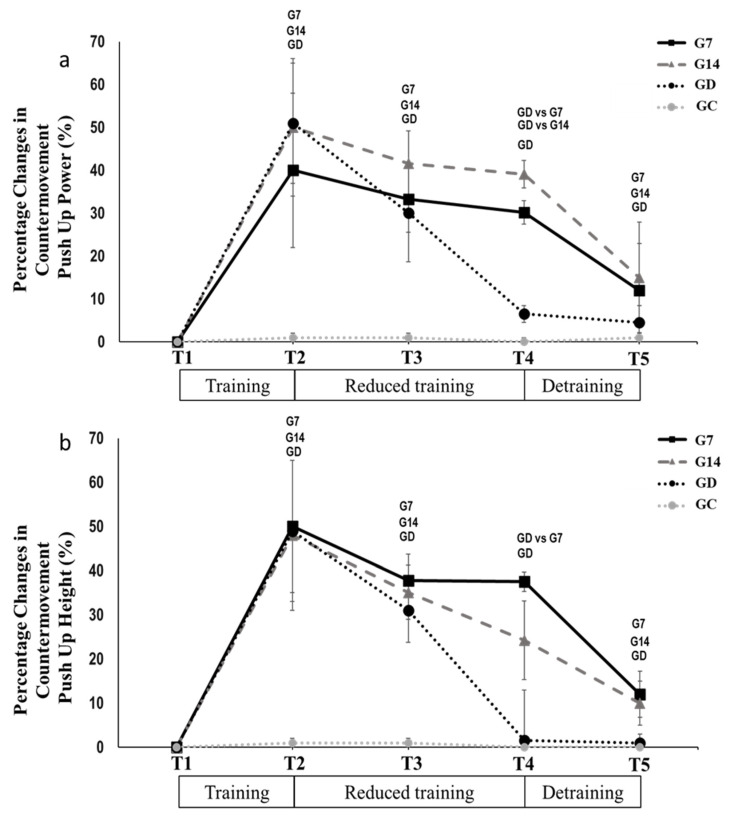
Percentage changes in countermovement push-up (**a**) power and (**b**) height, during all periods of the experiment. Small letters denote statistically significant differences in the marked group separately (where G7 = one training session every 7 days, G14 = one training session every 14 days, and GD = exercise cessation) between time periods (T1 to T2, T2 to T3, T3 to T4, and T4 to T5). When a comparison between groups (for example, GD vs. G7) is presented, it refers to the significant differences between the denoted groups in the marked time points.

*Upper extremities power performance:* A significant reduction of CMPU power and height was observed in all groups from T2 to T3. The lowest reductions were found in G7 (*p* < 0.05, Hedges’ g = 0.124; *p* < 0.05, Hedges’ g = 0.419; respectively, Table 2) followed by G14 (*p* < 0.05, Hedges’ g = 0.212; *p* < 0.05, Hedges’ g = 0.397; respectively, Table 2) while the greatest reductions were found in GD (*p* < 0.05, Hedges’ g = 0.984; *p* < 0.05, Hedges’ g = 0.920; respectively, Table 2). From T3 to T4, significant reductions were observed only in GD (*p* < 0.05, Hedges’ g = 1.608; *p* < 0.05, Hedges’ g = 1.475, respectively, Table 2), reaching the pre-training values. From T4 to T5, significant reductions were observed in G7 (*p* < 0.005, Hedges’ g = 0.298; Hedges’ g = 0.498, respectively, Table 2) and G14 (*p* < 0.05, Hedges’ g = 0.655; −13.2 ± 5.3%, Hedges’ g = 0.565, respectively, Table 2), with both groups reaching the pre-training values. The above parameters remained unchanged in GC (Figure 5).

**Figure 6:**
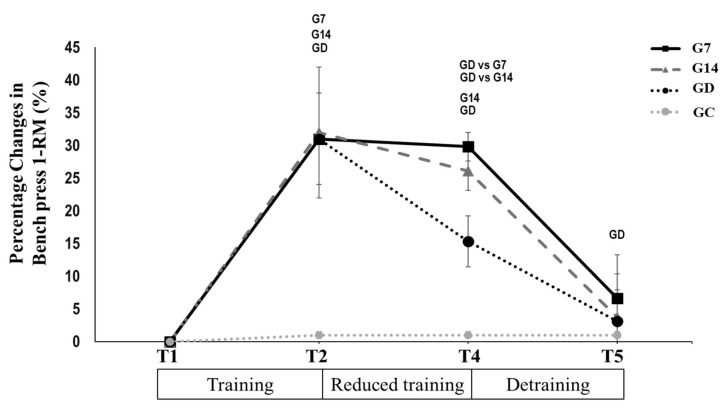
Bench press 1-RM percentage change after 12 weeks of systematic training (T2), after 12 weeks of reduced frequency training (T4), and after 12 weeks of detraining (T5) for G7, G14, GD, and GC groups. Small letters denote statistically significant differences in the marked group separately (where G7 = one training session every 7 days, G14 = one training session every 14 days, and GD = exercise cessation) between time periods (T1 to T2, T2 to T4, and T4 to T5). When a comparison between groups (for example, GD vs. G7) is presented, it refers to the significant differences between the denoted groups in the marked time points.

*Bench press 1-RM*: Maximum strength in G7 remained unchanged from T2 to T4 (*p* > 0.05, Hedges’ g = 0.228; Table 3). In contrast, it decreased significantly in G14 (*p* < 0.005, Hedges’ g = 1.161; Table 3) and GD (*p* < 0.005, Hedges’ g = 1.303; Table 3). During the period between T4 and T5, bench press 1-RM decreased in G7 (*p* < 0.005, Hedges’ g = 2.437; Table 3), G14 (*p* < 0.005, Hedges’ g = 2.567; Table 3) and GD (*p* < 0.005, Hedges’ g = 0.772; Table 3) reaching the pre-training values (Figure 6). There was no significant change for GC in any time period (*p* > 0.05).

*Cardiovascular performance:* Maximal aerobic power and heart rate at 100 and 125 Watts remained unchanged from T2 to T4 in G7 (*p* > 0.05, Hedges’ g = 0.096; *p* > 0.05, Hedges’ g = 0.362; *p* > 0.05, Hedges’ g = 0.178; respectively, Table 3). In contrast, they changed significantly in G14 (*p* < 0.05, Hedges’ g = 1.024; *p* < 0.05, Hedges’ g = 1.085; *p* < 0.05, Hedges’ g = 1.016, respectively, Table 3), while they returned to pretraining values in GD (*p* < 0.05, Hedges’ g = 1.231; *p* < 0.05, Hedges’ g = 1.606; *p* < 0.05, Hedges’ g = 1.288, respectively, Table 3). From T4 to T5, significant changes in maximal aerobic power and heart rate at 100 and 125 Watts were observed in G7 (*p* < 0.05, Hedges’ g = 0.696; *p* < 0.05, Hedges’ g = 0.529; *p* < 0.05, Hedges’ g = 0.493, respectively, Table 3) and in maximal aerobic power in G14 (*p* < 0.05, Hedges’ g = 0.978; Table 3), with both groups reaching the pretraining values. There was no significant change for GC in any time period (*p* > 0.05).

*Body Composition*: Lean mass of the upper extremities remained unchanged from T2 to T4 in G7 (*p* > 0.05, Hedges’ g = 0.102; Table 3). In contrast, it decreased significantly in G14 and GD (*p* < 0.005, Hedges’ g = 0.166; *p* < 0.05, Hedges’ g = 0.333, respectively; Table 3), with GD reaching the pre-training values. From T4 to T5, significant reduction occurred in G7 and G14 (*p* < 0.05, Hedges’ g = 0.250; *p* < 0.005, Hedges’ g = 0.166, respectively, Table 3) reaching the pre-training values. There was no significant change for the control group in any time period (*p* > 0.05).

**Figure 7:**
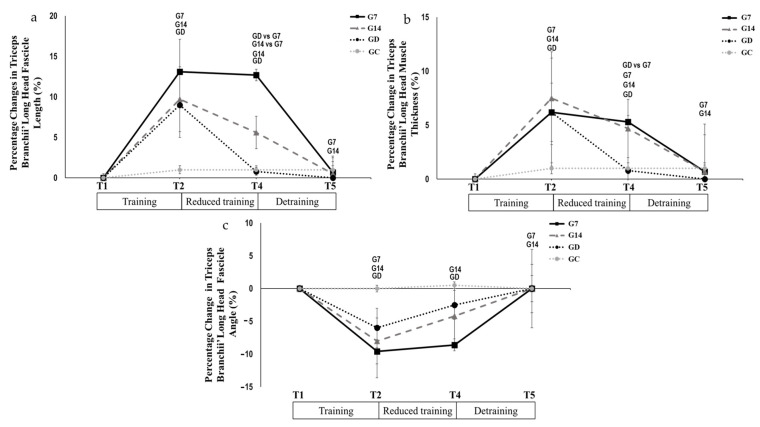
Percentage changes in triceps branchii long head (**a**) fascicle length, (**b**) muscle thickness, and (**c**) fascicle angle during all periods of the experiment. Small letters denote statistically significant differences in the marked group separately (where G7 = one training session every 7 days, G14 = one training session every 14 days, and GD = exercise cessation) between time periods (T1 to T2, T2 to T4, and T4 to T5). When a comparison between groups (for example GD vs. G7) is presented, it refers to the significant differences between the denoted groups in the marked time points.

*Triceps brachii muscle architecture*: Triceps branchii long head fascicle length (*p* > 0.05, Hedges’ g = 0.013; Table 3) and fascicle angle (*p* > 0.05; Hedges’ g = 0.044; Table 3), remained unchanged from T2 to T4 in G7. In contrast, they decreased significantly in G14 (*p* < 0.05, Hedges’ g = 0.392; *p* < 0.05, Hedges’ g = 0.202, respectively; Table 3), while they returned to pre-training values in GD (*p* < 0.05, Hedges’ g = 0.848; *p* < 0.005, Hedges’ g = 0.362, respectively, Table 3). During the period between T4 and T5, the above parameters, decreased in G7 (*p* < 0.005, Hedges’ g = 1.131; *p* < 0.005, Hedges’ g = 0.511, respectively, Table 3) and in G14 (*p* < 0.05, Hedges’ g = 0.676 and; *p* < 0.005, Hedges’ g = 0.190, respectively, Table 3), reaching the pre-training values, while they did not change any further in GD (*p* > 0.05, Hedges’ g = 0.095–0.054, respectively, Table 3). Triceps brachii thickness changed significantly from T2 to T4 in G7 and G14 (*p* < 0.005, Hedges’ g = 0.100; *p* < 0.05, Hedges’ g = 0.400, respectively, Table 3), while it reached pre-training values in GD (*p* < 0.005, Hedges’ g = 0.700; Table 3). During the period between T4 and T5, triceps brachii thickness decreased in G7 and G14 (*p* < 0.005, Hedges’ g = 0.600; *p* < 0.05, Hedges’ g = 0.600, respectively, Table 3), reaching the pre-training values. There were no significant changes (*p* > 0.05) for GC in the above parameters in any time period (Figure 7).

## 4. Discussion

The main finding of the present study was that performing one training session every 7 days or one training session every 2 weeks, during a 12-week period of forced reduced training frequency, leads to preservation of the vast majority of the upper extremities’ muscle power, muscle strength, and muscle architecture characteristics, without any significant differences between the two training frequencies. These observations indicate that both training frequencies are able to preserve 72–95% of training-induced adaptations during a 12-week period of forced reduced training frequency. Moreover, after 12 weeks of exercise cessation, all training-induced adaptations in both groups were lost. To our knowledge, this is the first study evaluating the effect of two different reduced training frequencies on muscle strength, power, and morphology in young females, using concurrent training, at which resistance training performed in the upper extremities and endurance training (high intensity cycling) in the lower extremities.

According to the results of the present study, a concurrent training incorporating power training for the upper extremities and HIIT aerobic training for the lower extremities, significantly promotes the upper extremities’ muscle strength, muscle power, and muscle mass adaptations, as well as the cardiovascular performance. The observed aerobic adaptations are in line with those reported in studies showing that the incorporation of resistance training does not negatively affect the adaptations related to aerobic power, at least in novice participants [9,38]. Furthermore, the present study provides strong evidence that supports the observation of a previous study, indicating that the addition of lower extremities aerobic exercise after resistance training for the upper extremities does not inhibit the upper extremities’ neuromuscular adaptations [39]. In contrast, it seems that when individuals perform concurrent training, which includes resistance and endurance training at the same muscle/muscle groups, especially when the concurrent training is performed from trained individuals/athletes, depending on the type, intensity, and volume of endurance training, the interference effect ranged from medium to high, and thus lower neuromuscular adaptations are observed [4,29,40,41,42,43,44,45]. Thus, according to the above and the results of the present study, it seems that there is not any interference effect after concurrent training at which power training and aerobic training (HIIT) performed in different muscle groups. Furthermore, considering the exercise order, it seems that performing resistance training before aerobic training (HIIT) does not attenuate the increases in muscle strength and mass. The observed results in this study are in line with those reported in studies showing that performing resistance training before aerobic training induces significant muscle strength adaptations [8,9]. On the contrary, performing aerobic training before resistance training, seems to lead to lower or no improvements in muscle strength, mass, and muscle power [27,28,46].

One concurrent training session every 7 days for 12 weeks preserved all the training-induced adaptations in triceps brachii fascicle length and angle and approximately 92% of muscle thickness. In contrast, one training session every 14 days for 12 weeks seems to induce significant reductions in triceps brachii fascicle length (−30%), angle (−40%), and muscle thickness (−28%) adaptations. In support of the present study results, engaging in high-intensity endurance and strength training once per week has been shown to lead to a 100% retention of adaptations in muscle mass and vastus lateralis muscle architecture and especially of muscle thickness, and only when this training stimuli was performed once every 14 days, significant reductions in muscle architecture/size were observed [7]. It is well known that even after a regular training period of frequent training sessions, power training leads to significant lower muscle hypertrophy than strength training [47]. Furthermore, as for muscle architecture properties, power training seems to induce increases in fascicle length, decreases in fascicle angle, and low or no significant changes in muscle thickness [29,37,48,49] adaptations, which seems to be dose-dependent to the imposed weekly training loads, with higher training loads inducing greater changes in muscle architecture properties [50]. Therefore, the significant reduction in muscle hypertrophy and muscle architecture properties, observed in the present study after the period of reduced training sessions, in the G14 group, could be attributed to the implementation of very infrequent power training sessions, which seems that they were not enough to provide the necessary stimuli required from the muscle to maintain its size and muscle architecture adaptations. This conclusion is further enforced by the fact that according to the existent literature, if the intensity and/or the volume of the training is maintained at the same level as those during the last training sessions of a regular/frequent training period, the training-induced adaptations will be preserved [7,23,25]. In the present study, we adapt these observations/propositions and kept them at the same level as those during the last training session. However, significant reductions were found both in muscle size and architecture properties in G14. Here, it must be pointed out that even if the adaptations in muscle size and architecture properties were reduced in the G14 group, participants of this group maintain 70–90% of their training-induced adaptations in muscle size and architecture properties, while the greatest preservations were observed with one training session per week. These findings are of high significance, as individuals who engage in such training once per week or even every 14 days, despite some negative changes in muscle mass and architecture properties, could be able to retain the vast majority of their training-induced adaptations. In contrast, as observed in the present study, complete detraining leads to the loss of all training-induced adaptations in muscle size and architecture properties. Taking into consideration the above, it seems that power training is not a potent stimulus which can be used during a period of forced reduction of very infrequent training (once per 14 days) for the maintenance of the training-induced adaptations in muscle size and architecture properties, and during such period, individuals should perform more frequent training session (such as once per week) to maintain most of the training-induced adaptations in their muscle hypertrophy and muscle architecture properties.

According to the results of the present study, it seems that muscle power decreases significantly if a training stimulus is provided every 7 or 14 weeks, at least for the first 6 weeks. Afterwards, it seems that muscle power is completely preserved at these levels even if a training stimulus is provided every 14 weeks, for at least another 6 weeks. Furthermore, muscle strength seems to have decreased significantly, only in the G14 group, after the end of the 12-week period of forced reduced training frequency. However, it must be noted that after both reduced training frequencies, the training-induced adaptations in muscle strength and explosive performance have not returned to pre-training values. Even if they were reduced, participants performing either one training session every 7 days or every 14 days seems that they lose only ~7% and ~9% of their training-induced adaptations in muscle strength and power, respectively, over a 12-week period of forced reduced training frequency. The above observations are of practical importance because as individual would lose only a very small proportion of their training-induced adaptations in their muscle strength and power, they could return to their regular intense/high volume training sessions and performances faster after a period of forced reduced training frequency. In contrast, again as it was observed in the present study, complete detraining leads to the loss of all training-induced adaptations in muscle strength and power. It has been recently reported that muscle strength could be maintained at the same levels as those observed after the end of a regular period of frequent training, if participants performed one concurrent training session (strength and HIIT endurance training) every week, for a period of 12 weeks [7]. In contrast, only when the concurrent strength and HIIT endurance training were performed once every 14 days, significant reductions in muscle strength were observed [7]. In line with these observations, in the present study, the G7 group seems to maintain maximum strength closer to that after the regular training period, while the greatest reductions were observed in the G14 group. Based on the above, it seems that muscle power and strength adaptations require different training frequencies to be maintained during a period of forced reduced training frequency.

An interesting observation was that the changes in muscle strength and power did not follow the same reduction patterns as those observed for muscle size/architecture properties, especially in the G14 group. Indeed, the magnitude of the reductions in muscle strength/power after the period of reduced training frequency were significantly lower than those of muscle hypertrophy/architecture properties, again especially in the G14 group. Thus, it seems unlikely that the changes in muscle power and strength could be attributed to the changes in triceps brachii size/architecture, and other biological factors may contribute to the group depending on preservation of muscle strength and power after a period of reduced training frequency. Power training leads to significant improvements of muscle power, mainly through very specific training-induced adaptation in neural system functioning [51] and muscle fiber composition (maintenance of type IIx muscle fiber and increases in type II and especially of Type IIx muscle fiber size and percentage cross sectional [52]. Moreover, it has been reported that the greatest increases in muscle power, which were accompanied by concomitant increases in percentage of cross-sectional area of vastus lateralis Type IIx muscle fibers, are observed, after a training period in which the weekly training loads were very low [52], almost at the same levels as those performing in the present study in the G7 group during the period of reduced training frequency. Thus, it could be hypothesized that the greatest preservations in muscle power, observed in the G7 compared to the G14 group, may be the outcome of the preservation of the vast majority of other biological adaptations like those in neural system functioning and muscle fiber composition, that contribute to power performance and adaptations, which probably decreased more in G14, and thus higher reductions in muscle strength/power performance were observed. However, as muscle strength and power performance were not returned to the pre-training values, in the G14 group, it could be hypothesized that even this training frequency is capable of maintaining most of the training-induced adaptations in neural system function and muscle fiber composition. Unfortunately, a limitation of the present study was that it was not feasible either to obtain muscle samples via biopsy or to evaluate the neural adaptations through electromyography during the training and detraining periods. Thus, the impact of the training-induced changes of the above parameters on the observed changes in muscle strength and power could not be evaluated, and future studies should investigate them.

One of the main goals of the present study was to identify which of the two training frequencies could serve as the minimum dose of training frequency that could lead to significant preservations of training-induced adaptations. According to the results of the present study, both reduced training frequencies lead to almost identical reductions in power performances (there were some greater decreases in the training-induced adaptations in the G14 group; however, they were not statistically different to those observed in the G7 group), compared to post regular training period values. Thus, we can conclude that if an individual/athlete at some point must undergo a period of reduced training frequency, by performing one training session every 14 days, they could preserve most of the training-induced adaptation in strength, power performances, muscle size, and muscle architecture properties. Thus, the minimum needed frequency, during such a period, could be proposed to be one training session every 14 weeks. However, a training frequency of one training session every week is needed to preserve the vast majority of muscle strength, architecture properties and size, training-induced adaptations, and to a greater extent, the training-induced adaptations in muscle power.

Lastly, after 12 weeks of detraining, the same results were found in both the G7 and G14 groups. In both groups, all muscle power, strength, muscle architecture, and aerobic adaptations were lost, a finding that was anticipated based on the findings of earlier studies [7,53,54]. The detraining period in the current study demonstrated the physiological and performance consequences of an insufficient training stimulus to maintain training-induced adaptations during at least 12 weeks of complete exercise cessation. It also showed that the training frequency during a period of forced reduction in training frequency has no significant impact on the magnitude or the rate of loss of the training-induced adaptations, during a period of complete training cessation.

## 5. Conclusions

The results of the present study suggest that muscle power/strength/mass and aerobic power decrease significantly, yet most of the resistance training-induced improvements are preserved with just six training sessions in 12 weeks (once every 14 days) after a period of systematic concurrent training. Therefore, performing one training session every 14 days for 3 months after a period of systematic concurrent resistance (upper extremities) and aerobic (lower extremities) training may preserve up to 90–95% of the muscle power, strength and up to 70% of muscle mass adaptations of the upper extremities, as well as up to 95% of aerobic power adaptations, achieved during the systematic training period if the training intensity and volume are sufficient. Nevertheless, if a person engages in a single training session each week, there will be significant reductions in power performance, reductions which do not have any significant differences with those observed after a training frequency of one training session every 14 days. However, one training session every 1 week seems to be needed in an effort to maintain the vast majority of the training-induced adaptations in muscle strength, muscle architecture, and size, at least for the initial 12 weeks of a period characterized by a mandated decrease in training frequency. The findings of the present study offer valuable insights for individuals aiming to sustain their adaptations in muscle power, strength, hypertrophy, and cardiovascular endurance, particularly those who encounter challenges in adhering to a regular training regimen for an extended duration. It is likely that maintaining a low training frequency, such as one session every two weeks, could facilitate a smoother transition back to a structured training program after a period of reduced frequency, resulting in minimal detriment to the strength/power adaptations achieved through prior training. On the contrary, an increased training frequency seems to be needed to maintain muscle architecture and size adaptations. Thus, coaches and athletes should use each one of these training frequencies, during a period of forced reduced training frequency, according to which adaptations they wish to maintain. Future research should further explore the role of upper extremities aerobic training (e.g., rowing machine ergometer) combined with upper extremities resistance training, on interference effect. Moreover, the role of different reduced training frequencies on the maintenance of the potential training-induced performance and muscle mass increases after systematic resistance and aerobic training for the upper extremities. Finally, future studies should also investigate the physiological–biochemical background of the observed results.

## Figures and Tables

**Table 1:** Body composition, vastus lateralis architecture, quadriceps cross-sectional area and performance, before and after the initial 12-week concurrent training period (T1 to T2).

		Training Group (N = 27)		Control Group (Ν = 7)	
		Before	After	Before	After
**Fat mass (kg)**		17.6 ± 4.2	17.9 ± 4.1	17.8 ± 2.8	18 ± 1.8
**Total LBM (kg)**		40.1 ± 2.5	40.3 ± 3.9	38.9 ± 2.5	38.6 ± 2.1
**LBM of upper extremities (kg)**		3.8 ± 0.5	4.1 ± 0.5 *^#^	3.7 ± 0.2	3.8 ± 0.3
**TB fascicle length (cm)**		6.2 ± 0.8	6.8 ± 0.9 *^#^	6.1 ± 0.1	6.1 ± 0.1
**TB fascicle angle (°)**		14.7 ± 2.5	13.5 ± 2.2 *^#^	14.9 ± 4.7	14.8 ± 4.7
**TB thickness (cm)**		1.3 ± 0.2	1.4 ± 0.2 *^#^	1.3 ± 0.2	1.3 ± 0.2
**CMPU power (W)**		127 ± 71	178 ± 77 *^#^	83.8 ± 17.2	80.4 ± 17.5
**CMPU height (cm)**		2.2 ± 1.1	3.4 ± 1.6 *^#^	1.4 ± 0.3	1.3 ± 0.4
**Maximal aerobic power (W)**		124.5 ± 22.4	148.0 ± 21.2 *^#^	117.2 ± 12.2	117.4 ± 12.7
**Heart rate**	**100 W**	153.1 ± 15.3	137.2 ± 14.1 *^#^	151.5 ± 8.2	155.4 ± 9.3
	**125 W**	166.5 ± 11.3	148.2 ± 10.4 *^#^	169.9 ± 11.2	173.5 ± 7.7
**1-RM bench press (kg)**		53 ± 6	70 ± 6 *^#^	52 ± 3	49 ± 5

Values are presented as mean ± standard deviation. Significant differences between T1 and T2 periods for each group are denoted by (*). Significant differences between groups for the marked time points are marked with (^#^) LBM = lean body mass, ΤΒ = triceps brachii, CMPU = countermovement push-up.

**Table 2:** Muscle power and height in countermovement push-up in G7, G14, and GD after 6 and 12 weeks of reduced frequency training (T2 toT3 to T4) and after 12 weeks of detraining (Τ4 τοT5).

	Τ2	Τ3	Τ4	Τ5
**G7 (N = 10)**				
**CMPU power (W)**	210.2 ± 96.1	198.3 ± 96.7 *	192.1 ± 94.1 *	163.4 ± 97.5 *^‡^
**CMPU height (cm)**	4.5 ± 1.9	3.8 ± 1.4 *	3.8 ± 1.3 *	3.1 ± 1.5 *^‡^
	**G14 (N = 10)**			
**CMPU power (W)**	175.5 ± 70.9	160.6 ± 69.1 *	156.5 ± 64.9 *	120.8 ± 41.5 *^‡^
**CMPU height (cm)**	3.7 ± 1.4	3.2 ± 1.1 *	2.9 ± 1.5 *	2.2 ± 0.9 *^‡^
	**GD (N = 7)**			
**CMPU power (W)**	137.1 ± 32.4	111.5 ± 17.1 *	84.2 ± 17.1 *^± G7 G14^	82.5 ± 18.1 *
**CMPU height (cm)**	2.9 ± 0.7	2.3 ± 0.6 *	1.6 ± 0.3 *^± G7 G14^	1.5 ± 0.3 *

With (*) denoting the significant difference within each group between the selected time points. (±) denotes the significant differences of the T4 period in relation to the T3 period for each group separately. (‡) denotes the significant differences of the T5 period in relation to the T4 period for each group separately. Small letters of the time points denote the significant difference between the marked time points for each group separately (where G7 = one training session every 7 days, G14 = one training session every 14 days, and GD = exercise cessation).

**Table 3:** Fat mass, lean body mass, lean mass of the upper extremities, triceps brachii architecture, and performance parameters in G7, G14, and GD after 12 weeks of reduced frequency training (T2 to T4) and after 12 weeks of detraining (Τ4 το T5).

		G7			G14			GD		
		**Τ2**	**Τ4**	**Τ5**	**Τ2**	**Τ4**	**Τ5**	**Τ2**	**Τ4**	**Τ5**
**Fat Mass (kg)**		15.63 ± 2.72	14.91 ± 3.12	15.76 ± 2.83	19.13 ± 4.46	19.40 ± 5.11	20.12 ± 4.71	18.62 ± 3.51	19.11 ± 3.91	20.12 ± 4.21
**LBM (kg)**		40.91 ± 3.92	40.91 ± 3.90	40.12 ± 3.83	41.62 ± 4.33	41.52 ± 4.11	41.3- ± 4.62	39.51 ± 4.82	39.31 ± 4.62	38.50 ± 4.52
**UE LBM (kg)**		4.21 ± 0.42	4.10 ± 0.40	4.01 ± 0.41 *^±^	4.11 ± 0.61	4.01 ± 0.62 *	3.91 ± 0.62 *^±^	3.91 ± 0.61	3.71 ± 0.62 *	3.62 ± 0.61 *
**Triceps Brachii Long Head**										
**Fascicle Length (cm)**		6.81 ± 0.84	6.84 ± 0.80	6.02 ± 0.61 *^±^	6.70 ± 0.91	6.41 ± 0.62 * ^G7^	6.03 ± 0.60 *^±^	7.01 ± 1.12	6.32 ± 0.60 * ^G7^	6.30 ± 0.91 *
**Fascicle Angle (°)**		13.01 ± 2.32	13.13 ± 2.25	14.11 ± 2.12 *^±^	13.90 ± 2.71	14.52 ± 3.21 *	15.17 ± 3.10 *^±^	13.71 ± 1.51	14.37 ± 1.81 *	14.62 ± 2.13 *
**Thickness (cm)**		1.41 ± 0.12	1.39 ± 0.13 *	1.33 ± 0.11 *^±^	1.51 ± 0.20	1.47 ± 0.21 *	1.41 ± 0.20 *^±^	1.55 ± 0.30	1.48 ± 0.37 * ^G7^	1.47 ± 0.31 *
**Bench Press**										
**1-RM (kg)**		75.21 ± 4.81	71.52 ± 3.91	54.84 ± 5.39 *^±^	73.20 ± 5.13	68.71 ± 2.01 *	53.46 ± 5.62 *^±^	64.61 ± 8.63	54.27 ± 7.31 * ^G7 G14^	48.28 ± 8.24 *^±^
**Cardiovascular Performance**										
**Maximal Power (W)**		147.51 ± 24.82	145.27 ± 22.95	125.32 ± 33.31 *^±^	157.54 ± 16.84	142.54 ± 12.14 * ^G7^	127.56 ± 18.01 *^±^	135.73 ± 15.02	114.29 ± 19.63 * ^G7 G14^	107.53 ± 26.71 *
**Heart Rate**	**100 W**	141.23 ± 13.27	146.17 ± 13.87	152.55 ± 10.13 *^±^	128.94 ± 16.07	142.72 ± 8.22 *	148.15 ± 14.35 *	145.16 ± 9.13	159.84 ± 9.26 *	162.82 ± 10.33 *
	**125 W**	160.21 ± 13.34	162.56 ± 12.27	167.51 ± 7.52 *^±^	148.41 ± 14.15	160.26 ± 8.21 * ^G7^	164.74 ± 6.87 *	156.33 ± 8.01	167.03 ± 8.64 * ^G7^	167.23 ± 4.03 *

(*) denotes the significant difference between T4, T5, and T2 for each parameter within each group. (±) denotes the significant differences in the T5 period in relation to the T4 period for each parameter within each group. Small letters denote statistically significant differences between the marked groups at each time point (where G7 = one training session every 7 days, G14 = one training session every 14 days, and GD = exercise cessation). UE LBM = Upper extremities lean body mass.

## Data Availability

Data are available upon reasonable request from the authors.
